# Evaluation of the Hierarchical Correspondence between the Human Brain and Artificial Neural Networks: A Review

**DOI:** 10.3390/biology12101330

**Published:** 2023-10-12

**Authors:** Trung Quang Pham, Teppei Matsui, Junichi Chikazoe

**Affiliations:** 1 Araya Inc., Tokyo 101-0025, Japan; trung_pham@araya.org; 2 Graduate School of Brain Science, Doshisha University, Kyoto 610-0321, Japan; matsui.teppei@gmail.com

**Keywords:** hierarchical correspondence, artificial neural networks, neuroscience

## Abstract

**Simple Summary:**

Artificial neural networks, inspired by the human brain, have demonstrated human-level performance across multiple task domains, raising the exciting possibility of them returning insights to neuroscientists about the human brain. However, artificial neural networks cannot be directly compared to the brain due to inherent differences in structure and computation. Here, we review the variety of approaches that researchers have thus far taken to evaluate the correspondence between the two, organized across multiple levels of analysis (node, layer, network, and behavior). In surveying these approaches, we note some of the insights uncovered, their limitations, and future directions in a domain of research that is developing quickly and with few established standards and practices. Our aim is to provide a systemized overview and guidance toward establishing a firmer theoretical and methodological framework in this emerging field.

**Abstract:**

Artificial neural networks (ANNs) that are heavily inspired by the human brain now achieve human-level performance across multiple task domains. ANNs have thus drawn attention in neuroscience, raising the possibility of providing a framework for understanding the information encoded in the human brain. However, the correspondence between ANNs and the brain cannot be measured directly. They differ in outputs and substrates, neurons vastly outnumber their ANN analogs (i.e., nodes), and the key algorithm responsible for most of modern ANN training (i.e., backpropagation) is likely absent from the brain. Neuroscientists have thus taken a variety of approaches to examine the similarity between the brain and ANNs at multiple levels of their information hierarchy. This review provides an overview of the currently available approaches and their limitations for evaluating brain–ANN correspondence.

## 1. Introduction

Understanding the information processing of the human brain is one of the biggest challenges for neuroscientists. In recent years, the artificial neural network (ANN) has become a powerful tool, performing at or better than human levels in several domains, including image classification (AlexNet [1]), conversation (ChatGPT [2], LaMDA [3]), games (Go [4], Starcraft II [5], and biological science (i.e., protein folding [6,7]). Growing interest has thus emerged as to the degree to which the information processing of ANNs can inform that which occurs in the brain.

Studies have shown that the processing of human perceptions is hierarchically distributed over the brain ([8,9,10,11,12,13]). In the visual domain, for instance, the V2 neuron appears to be sensitive to naturalistic texture stimuli [14], V4 neurons increase selectivity for the conjunction of features representing the surface shape (i.e., non-Cartesian gratings [15]), and IT neurons show stimulus selectivity, sensitive to the specific combinations of features, i.e., the face [16]. A similar hierarchy can be found in language processing [17,18,19], music processing [20,21,22], and tactile processing [23,24,25]. Taking a broader perspective, converging evidence alerts us to the brain’s global hierarchical organization beyond a collection of independent sensory hierarchies. At the cellular level, Murray et al. [11] found different decay rates of a single-unit activity in early sensory areas and distant cortical regions. Whereas sensory areas may need to respond more rapidly to environmental changes to reflect faster decay rates, regions involved in more complex, integrative cognitive tasks exhibit longer decay rates, suggesting a hierarchical ordering of a measure as intrinsic as single-neuron spiking timescales. Neuroimaging evidence of a global, sensorimotor-to-transmodal gradient supported this hierarchy of temporal dynamics, as well as other converging evidence such as increasing intracortical myelination, functional connectivity, and semantic processing along the gradient [26,27].

Given the intrinsic hierarchical architecture of ANNs, it becomes natural to wonder if they can capture the information processing that occurs in the human brain, thus serving as a framework for its understanding [28,29]. While both the human brain and the ANN are “black-boxes”, the latter is easier to customize and analyze. ANNs may provide a useful model for understanding the former, akin to how the atomic model can usefully convey the interaction between protons and electrons. As statistician George Box once said, “All the models are wrong, but some are useful”.

Relationships between the human brain and modern ANNs have been found since the early stages of ANN development. Studies have revealed the similarity between cognitive processing, such as vision and audition, and the hidden layers of ANNs [28,30,31,32,33,34,35,36]. The similarity was not limited to well-known “supervised” learning, but also “unsupervised” learning and “self-supervised” learning [37,38]. The growing number of studies in this area offers promise toward improving our understanding of the brain, as ANNs rapidly grow in sophistication and performance across problem domains. However, comparing ANNs to the brain to arrive at meaningful references is not a straightforward process. ANNs are inspired by the brain but are not replicas. Not only do they differ in substrate, but there are also vastly fewer ANN nodes than neurons. The principal algorithm that discovers the hierarchical circuitry of most modern ANNs is unlikely to exist in the brain [39].

Conventionally, evaluation of the similarity between the ANN and the human brain has been based on their performance in “intelligent” tasks (e.g., object detection, object classification, text generation, image generation, game playing, etc.). However, just this high-level comparison is inadequate for determining whether the ANN under the hood is undergoing comparable information processing to the brain. Neuroscientists have thus taken a variety of indirect approaches to evaluate the correspondence between ANNs and the brain. Here, we review the current approaches toward evaluating brain–ANN correspondence, considering their insights, limitations, and future directions.

## 2. Levels of Correspondence

Brain–ANN correspondence can be examined at multiple levels of the information hierarchy. A typical ANN consists of nodes and layers, where nodes constitute a layer, and layers constitute a network. These levels analogously map to neuron, region, and network levels of the brain.

A recent review of convolutional ANNs and human vision [40] considered two levels at which ANNs correspond to the brain: at the lowest level of neural activity and at the highest level of human behavior. But an examination of hierarchical correspondence would have to consider intermediate levels of hierarchy in the brain, such as those found in the visual system [28,31,32,33,34,35,36]. While the brain has served as inspiration for modern ANNs that achieve human-level performance, ANNs are not yet bound to the same global structural and functional hierarchy of the brain. We are indeed on the brink of large-scale multimodal ANNs (e.g., processing image, audio, and text); however, their emergence is driven more by engineering insights than natural selection or the neuroscience of brain correspondence. In other words, with the current and future likelihoods of divergence between ANN models and the brain, we will be better equipped to evaluate correspondence at multiple levels.

**Figure 1:**
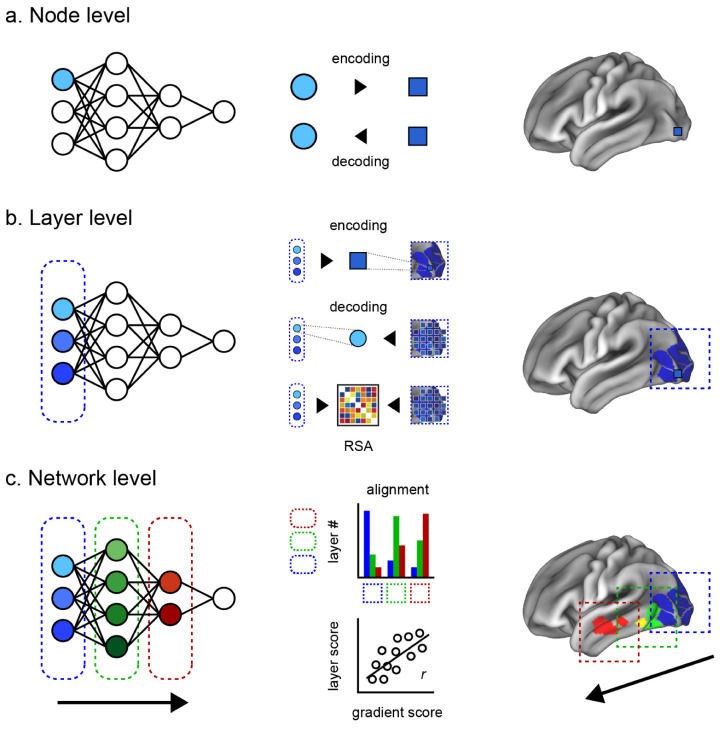

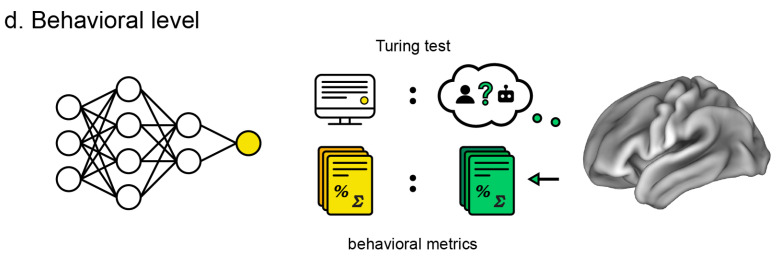
Four levels of correspondence evaluation. (**a**) The node level evaluates the correspondence between one node of the ANN and the smallest measured unit of the brain (e.g., a single unit recording, an electrode, a voxel). When computing correspondence via encoding, brain units are modeled using ANN nodes, and vice versa in decoding. (**b**) The layer level evaluates the correspondence between an ANN hidden layer and a brain region. Illustrated approaches show encoding via many nodes of a layer to a single voxel activity in a known brain region (**top**), decoding via many voxels to one node (**middle**), and correspondence between many nodes and many voxels via RSA (representational similarity analysis) (**bottom**). (**c**) The network level evaluates the correspondence between the overall information flow inside an ANN and across multiple regions, cortices, or the whole brain. Illustrated approaches sum the number of voxels in each region most associated with a given ANN layer (**top**), and examine the relationship between voxels’ scores (e.g., principal gradient score [26]) and their associated layers (**bottom**). (**d**) Behavioral level correspondence compares the ANN output, which includes qualitative assessments such as the Turing test in conversation, or compares the performance of behavioral metrics (e.g., classification accuracy, response time, error pattern, playing games) against their human counterparts. Brain illustrations were generated using the Connectome Workbench visualization software v1.4.2 [41] with Gordon’s parcellation [42].

Here, we introduce two intermediate levels toward a more comprehensive evaluation of the brain–ANN correspondence. Our review is thus structured along four levels of brain–ANN correspondence as follows (Figure 1a–d).

- Node level: Correspondence between one node of the ANN and the smallest unit of the brain (a single-unit recording, an electrode, or a voxel, depending on the measuring instruments).
- Layer level: Correspondence between an ANN hidden layer and a brain region (e.g., V1, V2, V4, IT).
- Network level: Correspondence between the overall information flow inside an ANN and inside the brain (e.g., hierarchical representation, multimodal network integration).
- Behavioral level: Similarity of ANN behavior, output, or performance metrics (e.g., image classification and generation, conversation, folding protein, and playing games) compared to the human counterparts.

## 3. Node Level

At the node level, correspondence is examined between one node of the ANN and the activity of a neuron, or the smallest unit of neural measurement, such as an electrode or a voxel. The idea is that if the information at one node can effectively predict the activity of a single neuron, or vice versa, it is likely that they are associated. Modeling can be bidirectional, where “encoding” refers to an ANN node predicting a neuron, and “decoding” refers to a neuron predicting a node.

### 3.1. Encoding/Decoding

In an encoding study, Yamins et al. [30] obtained the visual response of every node in a visual categorization ANN and used them to predict the neural recordings from V1, V4, and IT sites in rhesus macaques, collected via implanted multielectrode arrays. Linear regression was used for identifying the best combination of ANN unit outputs, ultimately accounting for more than 54% of the variance of the image-evoked brain responses. Bashivan et al. [32] used an ANN-driven image synthesis method to generate texture-like patterns that pushed the predicted spiking activity of neural sites (V4) beyond the range of naturalistic stimuli (a firing rate that was 39% higher than the maximal naturalistic firing rate). In human subjects, Zhuang et al. [37] extended the evaluation at node level correspondence along the ventral visual stream with an unsupervised learning method. Their ANN trained with unsupervised objectives using deep contrastive embeddings beforehand exhibited a neural predictivity comparable to its supervised counterpart.

Correspondence can also be demonstrated in the opposite direction, such as by Horikawa et al.’s [35] decoding approach, where a single node was predicted using voxel information from the visual cortex. Then, based on which features were predicted, the authors were able to identify which object categories participants saw or imagined. The predicted feature values were shown positively correlated with the true values across test images.

Studies outside the visual domain have illustrated the potential for creatively analyzing ANNs at the node level. In the auditory domain, Drakopoulos [43] used ANNs to model mechanical (inner-hair-cell transduction) and neural (auditory-nerve fiber) processing of sound that accurately simulated single-unit auditory nerve responses. Further, Nakai and Nishimoto [44] examined fMRI data of participants performing mathematical problems, training ANNs on distributed representations of quantity and mathematical operations that predicted activity in the intraparietal sulcus.

### 3.2. Limitations

An evident limitation here is interpretability, whether encoding or decoding. The single neural response is noisy and the weight of the encoder is not straightforward for interpretation [45]. Even in the ideal case where a node could perfectly model a neuron, the model would be meaningless without understanding what the ANN is trained to perform and in which functional brain region the neuron is situated. Once positioned in an appropriate framework, it can model and potentially facilitate insight into otherwise opaque information like neural activity in primates.

Another limitation of node-level evaluation is its scalability and difficulty of training. Comparison of every node in the deep neural network with the brain neuron becomes impractical with an increasing depth of the typical ANN. However, we note a collection of studies of spiking neural networks (SNNs), a type of ANN that takes a set of spikes as input and produces a set of spikes as an output that closely mimics the behavior of biological neurons [46]. The idea is that the temporal difference between spikes and their timing may contribute to the functionality. By turning the real-valued signals into binary ones, SNNs reduce the costs of signal transmission and computational complexity. While SNNs currently lag behind other ANNs in terms of performance, they highlight for us the potential for inventive ANN approaches in this domain.

## 4. Layer Level

Correspondence at the layer level examines the information within a hidden ANN layer to a brain region, such as V1 or IT cortex. Compared to the node level, the correspondence of a layer to a brain region can offer a better interpretability based on the well-established hypothesis of the brain region’s function, based on the accumulated history of anatomical and functional studies. However, we do not know the precise hierarchical boundaries of brain regions across the variety of information that it encodes, nor do we know whether or how these regions align with the relatively well-defined layers of an ANN. This has resulted in a variety of different approaches to evaluating the different possibilities of correspondence between different ANN layers against different brain regions.

### 4.1. Encoding/Decoding

Encoding and decoding approaches can be extended to the responses of a whole brain region instead of a single unit. Eikenberg [47] tested layer-level correspondence using a linear predictive model to encode each voxel’s activity in the human visual system based on reduced sets of layer features (e.g., subsampling ∼$10^{6}$ features down to ∼$10^{4}$ from the first ANN layer). They found a lower-to-higher-layer hierarchical mapping across dorsal and ventral visual stream regions-of-interest (ROIs). Two studies examined layer-level correspondence in non-human primates, applying encoding to single-unit activity. Cadena et al. showed the capacity for a pre-trained image classification ANN to predict V1 responses to images in rhesus macaques [48]. They found ANN correspondence to V1 spiking activity across multiple early hidden layers, and better than prior Gabor filter bank models, suggesting a more complex picture of nonlinear processing in early visual regions. In a creative encoding study employing different types of ANNs, Kar et al. [49] showed evidence suggesting that the ventral visual stream may not be just one-way hierarchical but may rely on recurrent, feedback connections. They modeled each electrode recording site in the V4 and IT cortex of rhesus macaques using layer nodes (linear combination of the first 1000 principal components), which showed that shallower recurrent ANNs could explain more variance in IT activity than “regular-deep” ANNs (8 layers), and comparably to deeper ANNs (more than 20 layers).

The visual system’s hierarchical correspondence across layers has been mirrored in decoding studies [33,34]. These findings were demonstrated by Horikawa and Kamitani, decoding not just images seen but also dreamt [34] and imagined [35], where decoding accuracy, evaluated by correlation between the category feature and decoded feature, was greater in deeper ANN layers and corresponded to higher visual cortical regions implicated in object recognition (lateral occipital complex, fusiform face area, and parahippocampal place area).

The limitations of these encoding/decoding approaches are their computational cost and the need for large amounts of training data, growing with the number of layers constituted inside an ANN.

### 4.2. Representational Mapping

The above techniques largely employed mapping many-to-one techniques, such as encoding many nodes of a layer to single-unit recordings in monkeys, then interpreting that activity in the context of a particular brain region. However, researchers may want to evaluate the many nodes of a layer to the many measured voxels of a region. A popular technique for evaluating the correspondence between the human brain and ANN that emerged from analyzing multivariate brain activity patterns [50] is representational similarity analysis (RSA) [51,52,53]. RSA facilitates the comparison of two disparate information sets by converting each set to a unitless space of “distances” that characterizes its geometry (i.e., a representational dissimilarity matrix; RDM) [52] in order to test the similarity of the two datasets’ underlying structures. This enables evaluating the correspondence of two datasets that differ in units and dimensions, such as computational models, EEG, MEG, fMRI, neural recording, or nodes in a layer versus voxels in a region [52], regardless of whether the data originate from across species, modalities, models, or condition [54].

For example, Cichy et al. [55] combined temporal (magnetoencephalography; MEG) and spatial (fMRI) information in response to images. They showed a hierarchical correspondence between ANN layers and human visual processing in early V1 to farther upstream areas both ventral (IT) and dorsal (intraparietal sulcus regions 1 and 2), with RDMs computed at each MEG frame yielding dynamic timecourses of ANN correspondence. Similarly, Khaligh-Razavi et al. [51] examined supervised and unsupervised ANN models to responses toward object images in human IT (measured via fMRI) and in monkey IT (measured via cell recording). Comparing RDMs, they found correspondence of human and monkey IT RDMs to those from the last, fully connected layers of supervised ANNs but not in any layers of unsupervised ANNs.

The use of RSA is not without limitations [56]. When exposed to novel stimuli, correspondence could be vanished. In RSA, scores can be a biased product of the structure of the dataset rather than the actual data [57] and may benefit from approaches such as feature- and voxel-reweighting [56] and voxel-wise-encoding RSA [38] to improve fit and correspondence. There are also structural concerns at the study level, such as those raised by Xu and Vaziri-Pashkam [58], who conducted an RSA-driven visual hierarchical correspondence study using block design fMRI and functionally defined ROIs (whereas prior studies [51,55] used a potentially noisier event-related design and anatomically defined ROIs). Using this approach, the authors evaluated 14 different ANN architectures and confirmed that lower layers of several ANNs could fully capture lower-level visual representations. However, none could achieve this for higher-level neural representations of real-world objects, suggesting the current limits of ANN–brain correspondence in visual representation.

## 5. Network Level

Network-level correspondence examines the overall information flow inside an ANN to a comparable network in the brain, such as the hierarchical representations across a single modality, or the multimodal integrative network across the whole brain. In relation to the layer-level correspondence, a straightforward approach is to quantify the alignment between the sequence of ANN layers and sequential processing expected in the brain. For example, one can compute the correlation between the two and count the nodes of layer that are most associated with each ROI in order to test if there is a shifting of distribution from low-level to high-level cortices. Given the intrinsic feed-forward characteristics of the ANN, a sequential alignment between the brain and the ANN would indicate a hierarchical network-level correspondence.

### 5.1. Sequential Alignment Approach

Early examinations of network-level correspondence have been conducted for sensory networks (visual network, auditory network) due to their interpretability [59]. For the visual network, the distribution of a model-explained variance of neural activity from Yamins et al. [30] shows a clear shift from V1 to IT as the layers changed from the first to the top layer. A similar correspondence across layers was found for information extracted along the visual ventral pathway [60] as well as the dorsal pathway [61]. A recent study from Mineault et al. [62] confirmed a similar correspondence between the ANN and the visual dorsal pathway in non-human primates. Using the ANN decoding approach, Horikawa and Kamitani showed that dreaming recruits visual feature representations that correlated hierarchically across the visual system [33]. For the auditory network, Kell et al. [36] found that an ANN trained on speech and music correlated with the auditory processing hierarchy in the brain with different layers processing different aspects of sound. In another study using ANNs trained to classify music genres, Guclu et al. [63] showed a representational gradient along the superior temporal gyrus, where anterior regions were associated with shallower layers and posterior regions with deeper layers.

Evaluating large-scale networks, such as across modalities or the global brain hierarchy, poses an additional problem. For instance, the actual hierarchical correspondence between the human auditory system and visual ANNs remains unclear, as other studies have raised the suggestion of parallel organization [64]. Spatial locations like brain coordinates may provide an intuitive correspondence but not concrete evidence of the brain’s structural–functional organization. For instance, not all of the many functional networks of the brain may adhere to a clear posterior-to-anterior hierarchy.

### 5.2. Gradient-Based Approach

**Figure 2:**
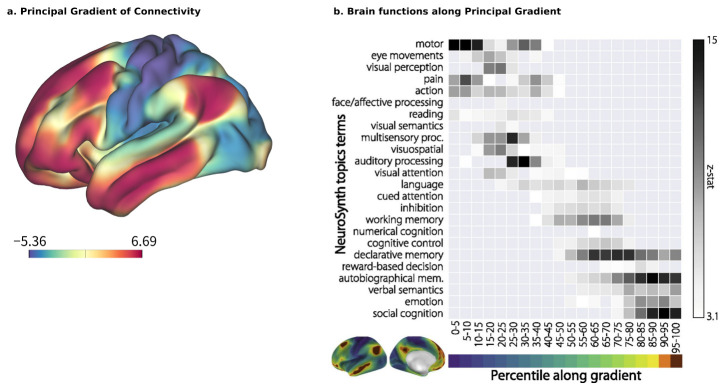
(**a**) Principal gradient (PG) of connectivity spans the brain, with lower scores at the start of the PG occupying sensorimotor regions and higher scores occupying higher cognitive and associative regions in a generally posterior-to-anterior gradient. (**b**) The distribution of brain functions along the PG begins at sensorimotor functions that converge transmodally toward higher cognition and affective processing. Figures adapted from [26].

Brain–ANN correspondence at the network level should also account for the sequence of chosen ROIs and the design of the ANN, such as the features that each node processes, whether they are processed sequentially or in parallel, how multiple modalities are integrated, and so on. A promising approach here is to use the principal gradient (PG) [26] as a reference. The PG is a global axis of brain organization that accounts for the highest variability in human resting-state functional connectivity (Figure 2a). Its arrangement begins with multiple satellites of unimodal sensory information that converge transmodally and integrate with the default mode network (DMN). A meta-analysis using the NeuroSynth database [65] has reinforced the relationship between cognitive function and position along the PG (Figure 2b), with sensory perception and motion exhibiting lower positions, and higher-order, abstract processes such as emotion and social recognition exhibiting higher positions [26]. The implications of PG on the hierarchical organization of functionality are further supported by clinical evidence, such as the compression of the principal motor-to-supramodal gradient in patients with schizophrenia (96 patients with schizophrenia vs. 120 healthy controls) [66] and the decrease in PG values in a neurodegenerative condition like Alzheimer’s disease [67].

For evaluating correspondence at the global brain level, the PG provides an independent, quantifiable metric of its hierarchy, anywhere from sensorimotor and transmodal to higher cognitive and affective information processing. Examining how subjective value emerges in the brain, ANNs individually trained to output subjective value from visual input have been shown to hierarchically correspond to the PG in the brains of those same individuals experiencing a similar value during fMRI [68], whereas Nonaka et al. [31] showed that most ANNs tend to have similar representations to the lower portion of the higher visual cortex (divided by the PG), but not the middle and higher ones, suggesting that findings of detailed correspondence in local areas could be more complex than simply an adherence to a global hierarchy.

## 6. Behavioral Level

The behavioral level is arguably the most important, as it serves as the entry point for ANN interest. It is the most important standard for commercial ANN use, but also critical for research since researchers ignored potential insights about the brain conferred by ANNs when they were not performing at human levels. At the same time, the behavioral level is the least scrutable. Usefully objective standards for similarity have been difficult to come by due to the open-ended nature of ANN domains and variance in their output.

### 6.1. Performance-Based Approach

Previous studies comparing ANN outputs directly to human performance showed that a higher categorization performance might be related to more explained variance at the high-level regions of the brain [30]. One may also consider other behaviorally relevant measures such as reaction times [69], error patterns [70], and out-of-distribution testing [71,72,73]. However, in more recent examinations, the most accurately categorizing ANNs do not necessarily compute the highest benchmark scores, such as brain score [74] and brain hierarchy score [31], which we will discuss in the next session.

More creative evaluations can be found in domains outside of sensory systems. For example, in language processing, Goldstein et al. [75] evaluated the three shared behavioral principles between the brain and ANN (called the deep language model): prediction before word onset, calculation of post-word-onset surprise from pre-onset predictions, and contextual embedding of words. While their results are supported by neural evidence derived from the recordings of electrocorticography (ECoG), employing similar principles does not assure similar hierarchical correspondence. Using fMRI, Caucheteux and King [76] also demonstrated a correlation between the true brain responses and ANN-predicted responses decreasing with the language performance.

Overall, a performance-based evaluation at behavioral level is currently underdeveloped and insufficient for providing comprehensive insight into brain–ANN correspondence.

### 6.2. Turing Test

In terms of a qualitative evaluation of behavior, perhaps the most considered has historically been the Turing test [77]. Here, the test of similarity is whether a person in conversation finds machine output indistinguishable from human output. This can extend beyond the conversational domain, such as the embodied Turing test, which challenges AI models to interact with the sensorimotor world at skill levels akin to their living counterparts [78]. However, as Searle [79] noted, a limitation is that the Turing test is about observation, not an actual measurement of “understanding” or “consciousness”, which further underscores the challenge of evaluating machine behavior for brain correspondence.

Interestingly, recent advances in large language models like ChatGPT [2] and LaMDA [3] appear to have either passed or sidestepped the Turing test. But beyond convincing contents of conversation, ChatGPT may not appear human because it replies too quickly, with too few apparent spelling or grammatical errors, or because we intuit the quality of its incoherence over time as different from our fellow humans. Machine performance has rapidly developed in other domains as well, including image generation (DALL-E by OpenAI), games closed and open (AlphaGo [4], AlphaStar [5]), and biological science (protein folding [6,7]). Once human performance within these domains were attained, questions of human similarity shifted smoothly from a basic “Turing test” to one of generality across domains or of increasing abstraction (e.g., reasoning and logic puzzles ConceptARC test [80]). For better or worse, these moving targets of behavioral similarity reiterate the complexity and open-endedness of brain–ANN correspondence at this level.

## 7. Composite Evaluation

While brain–ANN correspondence at each level may be evaluated independently, the ultimate goal is not to understand each level in isolation. Thus, studies have often examined more than one level of correspondence [30,33,34,37,48,51,60,61]. Recently, researchers have attempt to summarize brain–ANN correspondence in multiple levels as a single composite score.

For example, the brain score computed by Schrimpf et al. [74] is the mean of three scores: two at the node level (neural predictivity scores at V4 and IT) and one at the behavioral level. However, the brain score may not adequately describe the hierarchical correspondence between the information flow across an ANN’s layers and its analogous network regions of the brain. In a composite evaluation that includes intermediate levels, the brain hierarchy score proposed by Nonaka et al. [31] computes the degree of hierarchical homology across ANN’s layers and brain areas. This score utilizes both the encoding/decoding capability (node/layer level) and the Spearman rank correlation between the alignment of the ANN’s layer and brain areas (network level).

Composite scores can quickly convey an intuitive correspondence, especially when computed across many ANNs. However, due to the head start of image classification ANNs, such large-scale examinations have been limited to the correspondence of the visual ventral stream of the brain, owing to the current preponderance of image processing ANNs.

## 8. Summary and Outlook

In this paper, we focused on how researchers have approached the problem of empirically evaluating the hierarchical correspondence between ANNs and the brain, which is at a high level separate from reviews such as those charting the history of ANN development. Surveyed studies were organized into four levels of evaluation (node, layer, network, and behavioral levels) in an attempt to present the available approaches systematically. As a new field, the methodologies and analyses that researchers have taken have been more creative and less established as standards. Thus, our aim here was that such organization could serve in part as guidelines for future studies that may be contextualized in a firmer methodological framework. This domain of brain–ANN correspondence poses many challenges and opportunities for innovative solutions moving forward.

Our survey found that the layer-level and network-level evaluations have largely been confined within well-established sensory networks (i.e., vision, audition). We anticipate that this will shift as ANNs expand in function, size, and complexity. For example, recent ANNs have encroached into the somatosensory networks [81], as well as hippocampal and entorhinal systems [82]. With a growing industrial interest and development of multimodal ANNs, network-level evaluations should rise in prominence. Or, with greater interest in ANNs that model our emotional or value systems, evaluations may include a wider range of networks such as the default mode network and limbic network. This may in turn highlight global hierarchies such as the PG for the architectural inspiration. Recent studies have also extensively identified other functional connectivity gradients within the isocortex, at the cerebellum, and at the hippocampus (for details, see [83]). As ANNs grow in variety and complexity, researchers may come to rely on broader composite evaluations where experimental results from many laboratories are combined into a single suite, including all domains of information such as vision, language, and motor control. The growth of online repositories sharing neural data, such as OpenNeuro or Neurovault, may provide infrastructural support for such endeavors.

There is room for innovation nearer the structural foundations of ANNs as well. For example, currently available ANNs assign the same learning behaviors to all nodes, largely ignoring the anatomical and functional varieties of neurons, such as network differences in the wiring between networks or the different activities between cell types. Taking a broader structural perspective, recent studies [84] have also found that not only brain connectivity but the brain geometry also contributes to its functionality. Thus, future novel approaches may incorporate properties like spatial connectivity and geometry.

Conventional approaches employed correlation to evaluate hierarchical correspondence, which may be susceptible to an incorrect assumption that brains and ANNs are similar in variance and linearity. To overcome such limitations, one may consider using canonical correlation analysis (CCA [85]) or mutual information (MI [86,87,88,89]), both of which better explain non-linear relations. CCA finds the best correlation coefficient between two transformed variables, whereas MI of two random variables measures the mutual dependence between them (i.e., the amount of information obtained about one random variable by observing the other). Along with entropy, MI is an important concept in information theory [90], more closely resembling what we observe in the brain.

It should be noted that the correlation between the ANN and the brain need not be solely feed-forward and one-on-one. A single brain region may be associated with a stack of an ANN’s hidden layers and vice versa [48]. Sexton et al. [91] found in reassessing the previous studies [34,92,93] that the neural interface does not show the early-to-early and late-to-late pattern any more. The correspondence may be regulated by feedback/top-down processing. The current state-of-the-art feed-forward architecture is not robust to partial visibility, which could be caused by physiological delays [94]. The consideration of top-down processing may enhance the explainability and robustness of the ANN in future work. Another characteristic of the human brain that would be problematic in comparing the human brain and ANN is its connectivity and functional dynamics [95], which poses meaningful concerns such as individual flexibility in processing stimulus novelty and relevancy, particularly in the higher cognitive areas.

Optimistically, we are reminded that the arrow of progress can point in more than one direction. While the invention of human flight may have taken inspiration from the animal kingdom, the subsequent advancements in aerodynamics have cooperatively informed our understanding of both natural and mechanical systems in unforeseeable ways. Analogously, even as the industrial applications of ANNs appear to diverge from neuroscience, they continue to be inspired by the systems inside the brain, such as attention, working memory, episodic memory, and continual learning [96]. Thus, moving forward, brain-inspired ANNs may inform our understanding of intelligence and the brain’s information processing, which may in turn reinforce the development of novel, more efficient, and comprehensive ANNs.

## Data Availability

No new data were created or analyzed in this study. Data sharing is not applicable to this article.
